# Monitoring islet specific immune responses in type 1 diabetes clinical immunotherapy trials

**DOI:** 10.3389/fimmu.2023.1183909

**Published:** 2023-05-22

**Authors:** Sefina Arif, Clara Domingo-Vila, Emily Pollock, Eleni Christakou, Evangelia Williams, Timothy I. M. Tree

**Affiliations:** Department of Immunobiology, King's College London, London, United Kingdom

**Keywords:** immuno-monitor, ELISpot, multimer, immunotherapy, trial, T cell

## Abstract

The number of immunotherapeutic clinical trials in type 1 diabetes currently being conducted is expanding, and thus there is a need for robust immune-monitoring assays which are capable of detecting and characterizing islet specific immune responses in peripheral blood. Islet- specific T cells can serve as biomarkers and as such can guide drug selection, dosing regimens and immunological efficacy. Furthermore, these biomarkers can be utilized in patient stratification which can then benchmark suitability for participation in future clinical trials. This review focusses on the commonly used immune-monitoring techniques including multimer and antigen induced marker assays and the potential to combine these with single cell transcriptional profiling which may provide a greater understanding of the mechanisms underlying immuno-intervention. Although challenges remain around some key areas such as the need for harmonizing assays, technological advances mean that multiparametric information derived from a single sample can be used in coordinated efforts to harmonize biomarker discovery and validation. Moreover, the technologies discussed here have the potential to provide a unique insight on the effect of therapies on key players in the pathogenesis of T1D that cannot be obtained using antigen agnostic approaches.

## Introduction

Type 1 diabetes is an autoimmune disease in which islet specific T cells are thought to play a pivotal role in destroying insulin producing β cells (1). Immunotherapy clinical trials in type 1 diabetes is gaining momentum and as more and more trials are performed, there is a need for robust immuno-monitoring assays which are capable of detecting and characterising islet specific immune responses in peripheral blood.

This review will evaluate the use of established assays to monitor changes in islet specific immune responses and examine advanced multiparameter assays that give a greater insight into the functional immune response.

Islet-specific T cells serve as possible biomarkers and as such can guide drug selection and dosing regimens. Furthermore, patients can be stratified by analysing these T cell biomarkers which can then guide suitability for participation in future clinical trials. This underscores the need for robust assays to measure T cell responses in type 1 diabetes.

Assays designed to measure T cell responses in type 1 diabetes need to meet precise requirements. Firstly, they need to be able to monitor responses in peripheral blood as the target organ, the pancreas, cannot be biopsied. Secondly, they need to be sensitive enough to detect cells which occur at low frequency in the blood and have a low affinity TCR. Thirdly, T cells targeting islet antigens can also be detected in healthy controls, so a knowledge of their functional phenotypes is important. Finally, assays measuring T cell responses need to be able to work with low sample volumes as many clinical trials often involve paediatric samples. **Table 1** presents a summary of the advantages and disadvantages of experimental approaches which have been extensively used to identify and characterise antigen-specific T cells.

**Table 1:** Experimental approaches to measure antigen-specific T cells.

Assay	Feature/Parameter measured/Readout	Assay requirements	Advantages	Disadvantages
T cell spot-based assays (ELISPOT & FluoroSpot)	Cytokine secretion	15 x 10^4^ – 1 x 10^6^ PBMCs/condition	* Provides quantitative (number of secreting cells) and qualitative (type of response based on the cytokine secreted) information at a single cell level.	* Limited number of cytokines detected (currently up to four cytokines).
		24 – 72 hours incubation (2, 3)	* Can be performed directly *ex vivo* (no expansion required).	* Labour intensive (4).
			* Objective enumeration of cytokine-secreting cells using automated readers (although spots need to be validated by the human eye to exclude potential artifacts or spots which have not been accurately counted).	* Bulk culture of PBMCs which does not allow determination of the type of cytokine-secreting cell.
			* Cells can be harvested for downstream analyses (e.g., transcriptional profiling or T cell cloning) (5).	* Cannot assess the phenotype of antigen-specific cytokine-secreting cells.
			* Good reproducibility in individual or across different laboratories (6, 7).	* Requires *in vitro* culture.
			* Whole antigens and synthetic peptides (in single or peptide pool format) can be used for stimulation.	
			* Does not require previous knowledge of HLA restriction or epitopes targeted when whole antigens or overlapping peptides are employed.	
			* Highly sensitive (6–8).	
			* Can be performed with fresh and cryopreserved PBMCs.	
			* Validation and assay harmonisation have been conducted (7).	
			* Successfully employed in multiple laboratories (9–13).	
Dye-dilution assays (e.g. CFSE)	T cell proliferation	1 – 2 x 10^6^ PBMCs/condition	* Flow-cytometry based assay that provides quantitative information (number of cells proliferating) and enables phenotypic characterisation of antigen-specific T cells when additional cell surface and intracellular markers are used.	* Limited functional information (ability of cells to proliferate in response to antigen *in vitro*).
		7 days incubation (14)	* Can be performed directly *ex vivo* (no expansion required).	* Poor reproducibility due to variable background proliferation (14).
			* Can be combined with flow cytometric sorting allowing transcriptomic, TCR analyses and cloning of autoreactive T cells at a single-cell resolution (15)	* Each sample must be analysed individually.
			* Whole antigens and synthetic peptides (in single or peptide pool format) can be used for stimulation.	* Subjective gating.
			* Does not require previous knowledge of HLA restriction or epitopes targeted when whole antigens or overlapping peptides are employed.	* Sensitive to bystander proliferation (which may overestimate the number of antigen-specific T cells) or bystander suppression caused by regulatory cytokines (IL-10 and TGF-beta) masking cells capable of proliferating (8).
			* Easy to perform (8).	* Extrapolation required to calculate precursor frequency.
			* Can be performed with fresh and cryopreserved PBMCs.	* Requires *in vitro* culture.
			* Validation and assay harmonisation have been conducted (6)	
			* Successfully employed in multiple laboratories (16–18)	
Peptide-MHC multimers	Frequency of epitope-specific T cells	1 – 2 x 10^6^ PBMCs/condition (HLA-class I)	* Allows detection and phenotyping of antigen-specific T cells directly *ex vivo* and estimation of their frequency. However, some studies claim *in vitro* expansion is required to increase the assay detection limit which, consequently, could alter the phenotype of these cells (19)	* Isolation and enumeration are challenging as this assay relies on low-affinity pMHC-TCR interactions. Standard pMHC tetramer staining is the most used method despite recent evidence demonstrating that it fails to detect antigen-specific T cells with very low affinities. The use of optimised protocols has been shown to increase the assay detection limit with results comparable to parallel functional assays (20).
		10 – 20 x 10^6^ PBMCs/condition (HLA-class II)	* Physical detection of antigen-specific T cells does not rely on a particular effector function, and therefore is not influenced by sample preparation or cryopreservation methods (20).	* Limited reproducibility across different laboratories (21, 22).
		No incubation required (20)	* The use of higher order multimers enhances the ability to recover a greater number of antigen-specific T cells (especially those with low-affinity TCRs) (20, 23, 24) and simultaneous detection of multiple epitope-specific T cells is possible using combinatorial MHC multimers (19, 25, 26).	* Does not provide functional information, although flow cytometric sorting combined with RNA transcriptional profiling enables characterisation of functional phenotypes.
			* Can be combined with magnetic bead separation or flow cytometric sorting for isolation of peptide-specific T cells (20).	* Relies upon HLA-restricted presentation of selected β-cell epitopes and therefore requires previous knowledge of the HLA type of the individuals assessed and epitopes targeted.
			* Compatible with enrichment methods to enhance detection of rare cell populations (27).	* A limited repertoire of epitope specificities can be tested in a single biological sample due to limitations in the number of available fluorochromes (25).
			* Good reproducibility in individual laboratories (21, 22).	* Subjective gating.
			* Allows identification of epitope specificities.	* Labour intensive.
			* Does not require *in vitro* culture.	
			* Can be performed with fresh and cryopreserved PBMCs.	
			* Validation and assay harmonisation have been conducted (22, 28)	
			* Successfully employed in multiple laboratories (23, 27, 29).	
Activation-induced marker (AIM) assay	Frequency of antigen-specific T cells	5 – 10 x 10^6^ PBMCs/condition	* Detection of antigen-specific T cells based on upregulation of TCR stimulation-induced surface markers by flow cytometry.	* Lack of sensitivity due to variable background response.
		16 – 24 hours incubation (30)	* Phenotype-agnostic method which allows identification of antigen-specific T cells with a variety of effector or regulatory functions (30–32).	* Does not provide functional information.
			* Additional information about responding T cells can be obtained using multiparameter flow cytometry.	* Subjective gating.
			* Can be performed directly *ex vivo* (no expansion required).	* Labour intensive.
			* Compatible with enrichment methods to enhance detection of rare cell populations (33).	* Requires previous knowledge about the kinetics of the AIM markers used.
			* Can be combined with flow cytometric sorting for isolation of antigen-specific T cells (33, 34). and downstream applications (e.g., single-cell transcriptional profiling).	* An accurate set of AIM markers is required for the identification of the complete pool of antigen-specific T cells.
			* Detection of responses to a wide array of antigenic targets, including whole antigens and synthetic peptides (in single or peptide pool format).	* Validation and assay harmonisation initiatives are lacking.
			* Does not require previous knowledge of HLA restriction or epitopes targeted when whole antigens or overlapping peptides are employed.	
			* Can be performed with fresh and cryopreserved PBMCs.	
			* Successfully employed in multiple laboratories (34–38).	

## ELISpot and fluorospot assays to measure antigen-specific responses

‘Spot’ based assays can detect a wide range of cytokines and are thus commonly used to evaluate functional T cell responses both in research and as diagnostic tools (39–44); they have been used in clinical diagnostic settings. These assays are quantitative, highly sensitive, can measure low-frequency antigen-specific T cells (detection limit 0.0001%) (45) and now are increasingly used in clinical trial monitoring in a variety of settings (46–50). Spot based assays such as ELISpot assays are functional assays and have been used in clinical diagnostic settings such as in tuberculosis diagnosis (51). In type 1 diabetes, these spot-based assays have been pivotal in understanding immune responses (9, 52–56).

**Figure 1:**
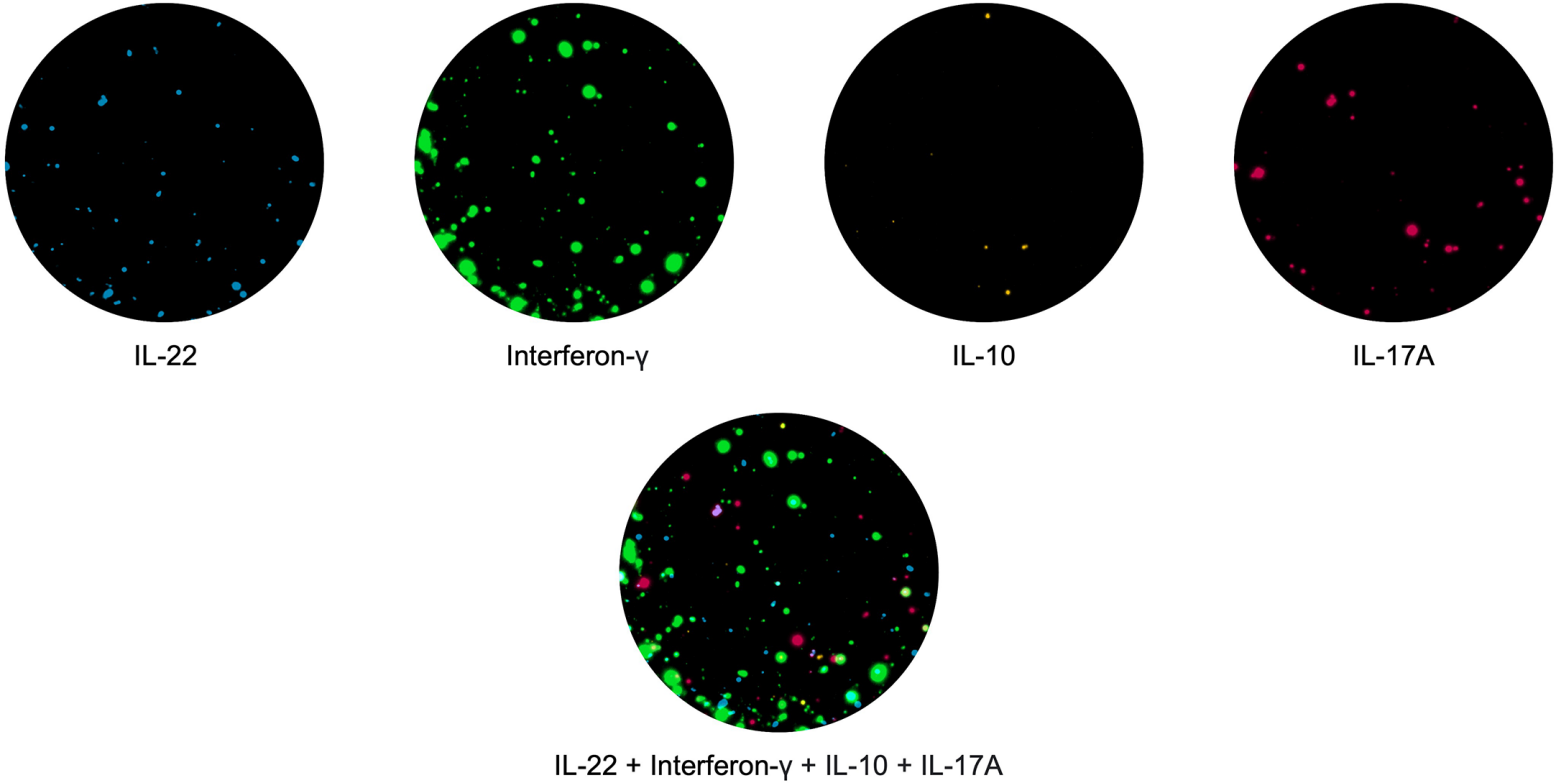
PBMCs were stimulated with the hexavalent vaccine, Infanrix and Candida albicans, and IL-22 (blue), interferon-γ (green), IL-10 (yellow) and IL-17 (red) T cell responses were measured. T cells making all 4 cytokines are shown in white.

In the past few years, the spot-based assay has been further modified using fluorophores attached to the secondary detection antibodies, this assay known as Fluorospot allows the simultaneous detection of T cell subpopulations with distinct cytokine profiles (57). This also has advantages over the ELISpot assay, for example, all cytokines are detected in the same well and therefore fewer PBMCs are needed; it is multiparametric, allowing enumeration of cells which secrete more than one cytokine at a given time (called double or triple secretors). Recently, Fluorospot assays measuring T cells co-producing up to 4 different cytokines have been described ( (58); Jerram et al., manuscript submitted; Domino-Vial personal observations)). (**Figure 1**).

### Monitoring immune-intervention studies with ELISpot assays

Mechanistic assessment of immune therapies can add a unique insight into understanding clinical trial outcomes. This may be particularly important when trying to interpret trials that have failed to meet their clinical end point. For example, a trial of nasal insulin was started with the idea of reducing pathogenic immune responses to insulin (59). Patients within 1 year of diagnosis self-administered nasal insulin (at a dose was 1.6mg) or placebo (insulin diluent) in the form of a spray daily for 10 days and then weekly on 2 consecutive days for 12 months. Patients were assessed at 3 monthly intervals for 24 months.

The trial failed to meet its clinical end point but suppression of antibody responses to subcutaneous insulin was observed, and in a subgroup of patients, there was also suppression of proinsulin-specific interferonγ T cells as demonstrated using an ELISpot assay. For technical reasons, proinsulin rather than insulin was used in the ELISpot assay, which means that responses could be targeted at epitopes present in proinsulin that are absent in insulin.

Together these data imply that tolerance was induced, providing a rationale to further pursue this strategy in at risk individuals (59).

Another trial that failed to meet its clinical endpoint was the GAD-alum trial (60). GAD65 is a commonly targeted autoantigen in type 1 diabetes, it was combined with alum as an adjuvant and subcutaneously administered to recent-onset patients with type 1 diabetes with the aim of shifting the autoimmune response from a TH1 to a TH2 response. Patients were given 20 µg GAD-alum (thrice in one cohort and twice followed by alum in a second cohort) or 3 injections of alum over 4-12 weeks and observed for a year after which it was clear that GAD-alum treatment did not show any insulin preservation (60). The trial failed in meeting its clinical endpoint but as demonstrated by IL-13 ELISpot, there was a clear induction of GAD-specific TH2 responses in a substantial proportion of patients, indicating a powerful immunological effect (61).

In other instances, mechanistic insights can validate clinical outcomes as shown in the proinsulin peptide C19-A3 trial. Patients with type 1 diabetes treated intradermally with 10µg of peptide C19-A3 either every 2 weeks or every 4 weeks (alternating with placebo) or with placebo (saline) over 6 months, showed maintenance of C-peptide levels. This was accompanied by peptide-specific IL-10 responses, measured by ELISpot, indicating that an immunoregulatory mechanism may be at play (62). In a follow-up study, using a mixture of six HLA-DRB1*0401 selective proinsulin and IA-2 peptides, C-peptide levels were preserved in half of the treated patients compared to none of the placebo treated. Immuno-monitoring in these patients using ELISpot assays, showed higher levels of antigen-specific CD4 T cells producing IL-10 and IL-17 in the treated patients indicating immunomodulatory mechanisms may be in operation (63).

ELISpot assays are useful in antigen-specific immunotherapy as they can measure cytokine responses directly to the antigen used in the clinical trial, unlike in non-antigen specific intervention strategies where the effect is generally more systemic. Furthermore, as an immune-monitoring tool, the ELISpot assay can monitor the efficacy of antigen-specific immunotherapy as demonstrated in a study designed to induce gluten-specific tolerance in patients with celiac disease (64). Patients were given gluten protein encapsulated in nanoparticles (TAK-101) or placebo by intravenous infusion and interferon-γ T cell responses to gliadin were measured by ELISpot at baseline and post therapy. In the treated patients, interferon-γ T cell responses to gliadin were reduced by nearly 90% suggesting that gluten-specific tolerance was induced by TAK-101 (64). In this setting, the ELISpot assay measured the efficacy of tolerance induction, however the method did rely on the need for *in vivo* gluten challenge which is not possible in type 1 diabetes.

### Monitoring immune-intervention studies with Fluorospot assays

The GAD specific TH2 response elicited by GAD-alum described above was further dissected using a Fluorospot assay which can measure cells co-producing different cytokines. It was observed that GAD specific T cells were bifunctional, producing both interferon-γ and IL-13. It is possible that these bifunctional cells were not able to sufficiently deflect the pathogenic TH1 response and this could explain the lack of clinical efficacy observed with GAD-alum (61). This is further supported by data from a preceding trial in which TH2 cytokines were detected in patients give GAD-alum but interferon-γ and IL-17 were also observed (65).

Spot based assays are also useful in interventions targeting relevant T cell derived proinflammatory cytokines in type 1 diabetes. Ustekinumab targets both IL-12 and IL-23 cytokines thereby blocking the induction of Th1 and Th17 cells respectively (66); it was used recently for the first time in a small cohort of newly diagnosed type 1 diabetes patients (67). This was primarily a safety and dose-finding study where doses of either 45mg or 90 mg were administered subcutaneously; decline in C-peptide levels were lowest at the higher dose of ustekinumab. Immuno-monitoring was conducted using a Fluorospot assay which showed a significant reduction in proinsulin specific interferon-γ and IL-17 T cells at the 90 mg dose but not at the 45 mg dose interestingly, GAD65 specific responses were not reduced at either dose. Thus, in this case, immuno-monitoring served to guide future dose selection. In terms of the mechanistic effect of ustekinumab, the Fluorospot assay demonstrated that pathogenic Th1 and Th17 cells were being restrained.

### Cryopreserved cells in Spot assays

Both ELISpot and Fluorospots have been extensively used in immuno-monitoring studies in type 1 diabetes and have provided valuable insights into mechanisms underlying therapeutic interventions. These assays have used both fresh and cryopreserved PBMCs with the latter having the advantage of requiring less manpower, reducing day to day variation, and, making it possible to test all time points on the same day. On the downside, there is a loss of cell viability, loss of cell subtypes, spontaneous secretion of some cytokines (21), and loss of specific signals such as IL-10 and IL-4 responses in ELISpot (68).

There is evidence to suggest that IL-10 is especially vulnerable to cryopreservation (69) and this needs to be considered when the mechanism of intervention may be regulatory.

## Proliferation assays to assess antigen-specific T cell reactivity

Proliferation assays have been extensively used in type 1 diabetes for decades to monitor immune responses. PBMCs are stimulated with antigen and proliferation tracked either by the incorporation of radiolabeled nucleoside (tritiated thymidine) or by the dilution of a florescent tracker dye (carboxyfluorescein diacetate succinimidyl ester (CFSE), cell trace violet or equivalent).

In a pilot trial, which evaluated subcutaneous versus intra-lymphatic GAD-alum administration in patients with type 1 diabetes, proliferation assays using radiolabeled thymidine showed reduced proliferation to recombinant human GAD65 in those patients given 4 µg GAD-alum intra-lymphatically compared to patients receiving it subcutaneously at 20 µg (70). The authors also reported a concurrent TH2 IL-13 dominated response in subjects with the better clinical responses implying tolerance induction. A recent follow-up study examining immune responses in these two groups after 15 months used proliferation assays with PBMCs stimulated with CD3/CD28 beads to show enhanced proliferation in most of the patients treated with intra-lymphatic GAD-alum compared to the subcutaneously treated patients (71); this also corresponded to significantly lower levels of HbA1c in the former indicating a better clinical outcome. Other parameters such as GAD autoantibodies and GAD induced cytokines also differed between the two groups demonstrating that the route of administration affects the resulting immune response.

Another trial using proinsulin peptide pulsed tolerance inducing dendritic cells (tolDCs) investigated immunological efficacy by using proliferation assays (72). Patients with long standing type 1 diabetes were given 2 escalating doses of proinsulin peptide C19-A3 pulsed tolDCs intradermally or saline (the tolDC vehicle) for one patient in each cohort and monitored for 6 months (73). Proliferation assays demonstrated reduced responses to proinsulin peptide C19-A3 in a third of patients and to whole preproinsulin in all the patients at 6 months with this persisting in the latter in some patients. The reduced proliferative responses were accompanied by an increase in a Treg subset (72), which supports the hypothesis that tolDCs polarize towards a regulatory T cell response (74). Such reduced proliferative responses upon antigen specific immunotherapy have been demonstrated in other settings including multiple sclerosis where autoantigenic peptides coupled to PBMC were used to induce antigen specific tolerance (75).

Proliferation assays in which PBMCs are labeled with fluorescent based dyes such as CFSE are becoming much more common in immuno-monitoring studies (76).

Using this technique PBMC proliferative cell responses were assessed in sequential samples from autoantibody-negative children at high risk of type 1 diabetes recruited into an oral insulin trial (Pre-POINT) (77); children were administered placebo or assigned in blocks to receive insulin at escalating doses from 2.5–67.5 mg. It is thought that oral administration of antigen could promote regulatory T cells (78). The cells proliferating to insulin were subsequently stained with a panel of antibodies and proliferating CD4+ T cells were single cell sorted and gene expression profiles analyzed. The data showed that Pre-POINT administration in high-risk individuals resulted in regulatory, insulin responsive cells. This approach allowed the authors to probe the response more extensively than using proliferation alone.

## Multimer assays to evaluate antigen-specific T cell frequencies

MHC multimers consist of complexes of HLA molecules combined with a specific peptide and conjugated to a fluorochrome (79) or rare metal ions which can then be detected using conventional flow cytometry (80) or mass spectrometry (81) respectively. MHC multimer technology has been shown to be an effective tool in the detection of rare antigen specific CD4 and CD8 T cells in type 1 diabetes (23, 82–85). These enumerating assays can be combined with downstream processes to phenotype cells (86, 87), or gain insights into TCR sequences (88).

In type 1 diabetes clinical trials, MHC multimer technology has been used in several trials (89–91). For example, it has been used to monitor, CD4 T cell responses in GAD–alum treated patients; newly diagnosed patients were given either one of 2 doses of GAD-alum or alum as a placebo and immune responses were monitored (90). The frequency of CD4 T cells recognized by GAD peptide loaded tetramers was increased in GAD-alum treated patients compared to those given placebo; subsequent phenotyping identified these cells as effector memory cells suggesting that this was a recall response where the cells were activated specifically in response to GAD-alum (90).

Standard MHC multimer technology uses tetramers loaded with a single peptide which can be limiting when monitoring multiple epitope specific T cell populations. This can be circumvented using a combinatorial labelling approach, allowing parallel assessment of multiple specificities through the use of individual tetramers labelled with more than one marker (fluorochrome or rare element) thus facilitating the detection of a large number of epitope specific T cells (>25) (92) in a single clinical sample (93). This approach can be successfully applied to antigenic peptides presented by both HLA class I and class II molecules, and it has recently been shown to provide a more informative and discriminatory measurement of islet specific CD8+ and CD4+ T cell frequency, respectively (24, 82).

This technique was used to investigate the effect of administration of a plasmid encoding proinsulin (BHT-3021) on islet specific CD8 T cells with a range of antigen and epitope specificities (89). Patients were given either a BHT-placebo or BHT-3021 at 4 different doses (ranging from 0.3 mg to 6 mg) intramuscularly for 12 weeks. C-peptide together with antigen-specific CD8 T cell responses to several islet antigen peptides were measured thereafter. The authors reported an increase in C-peptide in the BHT-3021 treated group compared to placebo with a concurrent decrease in proinsulin specific CD8 T cells (89).

Similarly, a trial employing autologous hematopoietic stem cell transplantation (AHSCT) where recent-onset type 1 diabetes patients were given an infusion of autologous hematopoietic stem cells used this technique to show that better clinical outcome was linked to baseline autoreactivity. The levels of islet antigen specific CD8 T cells did not change upon AHSCT but higher C-peptide levels and a longer diabetes free period were observed in patients with low CD8 islet autoreactivity at baseline prior to AHSCT (91).

## Activation induced marker assays

Activation Induced Marker (AIM) assays are used to study antigen reactive T-cells. AIM assays identify responding cells based on upregulation of cell surface markers associated with TCR engagement following incubation of PBMC with recombinant antigen or peptides. In theory, these assays make little prior assumption about the phenotype of responding cells, which can be captured for downstream analysis using methods such as single cell RNA-Seq (scRNA-Seq). The most common version of this assay uses upregulation of CD69 and CD154 to identify antigen-specific T cells (31) and has been used to study islet specific T cell responses (33, 94).

AIM assays have been used for monitoring in intervention trials in T1D (95); they are highly informative in both identifying the phenotypes of responding T cells and in providing an insight into quantitative changes in distinct cell populations. This has been demonstrated in the aforementioned intralymphatic GAD-alum trial where administration resulted in an immunomodulatory response; phenotypic profiling of the immune cells showed an induction of PD-1+ T follicular helper cells and exhausted CD8 T cells (95).

### Activation induced marker assays and single cell transcriptional profiling

Although the inclusion of additional surface or intracellular markers in an AIM assay does provide additional information about the phenotype of the responding cells, unbiased analysis techniques may provide a greater understanding of the mechanisms underlying immuno-intervention. This can be achieved by the combination of AIM assays with single cell transcriptional profiling such as using plate-based methods (sorting directly into 96-well plates, e.g., SMART-seq), droplet-based technologies (e.g. 10x Chromium) or cartridge-based techniques (e.g. BD Rhapsody). Each technology has strengths and limitations as discussed elsewhere (96). The 10x Genomics platform has the advantage over both the plate- and cartridge- based platforms in that it can capture and process high numbers of cells in a single experiment (high-throughput sequencing) (97) however, it does incur cell loss (up to 50%) (98, 99) In contrast, cell loss is lowest when using plate-based methods, but this is significantly more expensive and time consuming (96, 100).

**Figure 2:**
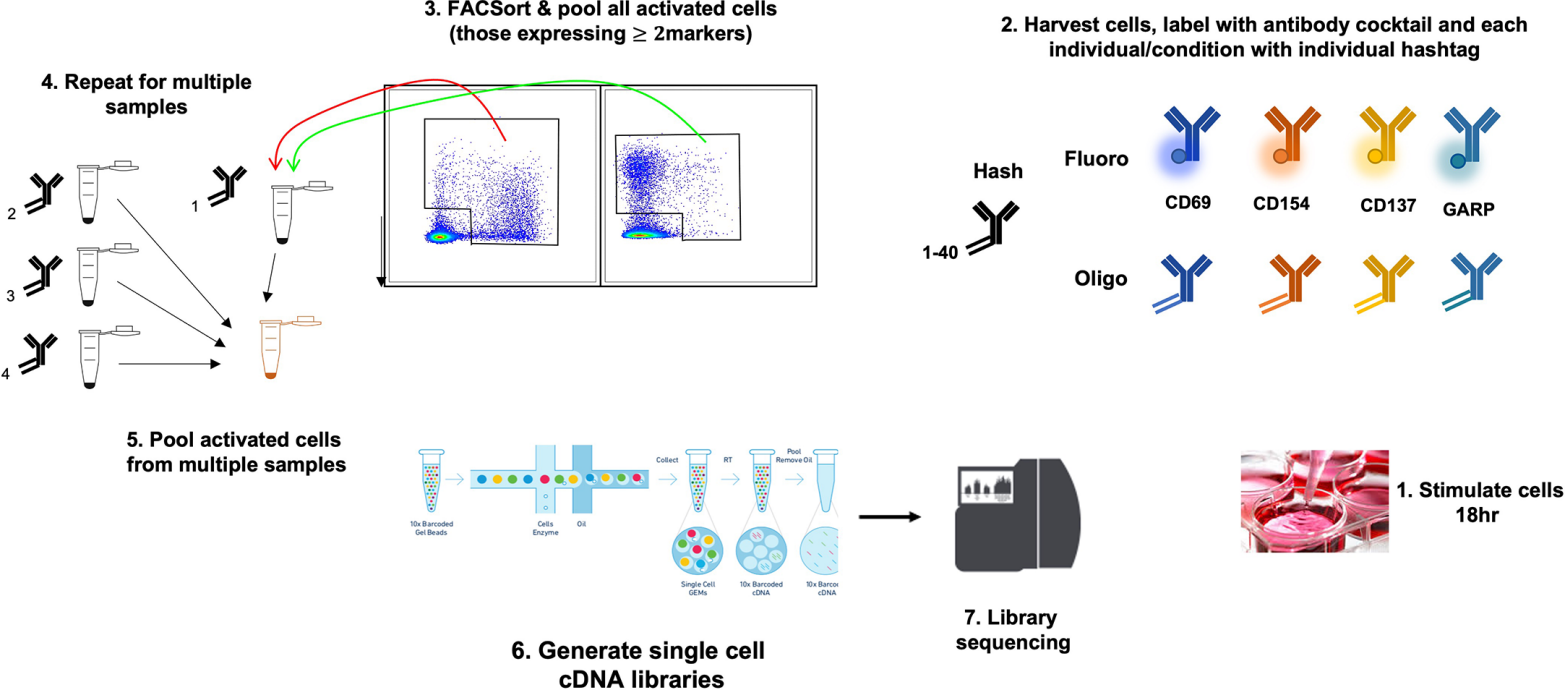
Use of the AIM 10x assay to investigate islet-specific T cells. A schematic diagram showing combination of AIM and s.c. RNA-Seq to characterize antigen-specific T cells.

This has been tested on PBMCs stimulated with GAD and proinsulin from patients with type 1 diabetes and healthy controls, and in each case, 100-500 autoantigen specific cells were profiled; these were subsequently identified using cell surface markers and further characterized by examining differentially expressed genes within the various cell populations (E. Christakou et al., manuscript in preparation) (**Figure 2**).

Although the cost of AIM assays combined with single cell RNA-Seq can be prohibitive when testing numerous clinical trial samples, barcoding technology allows samples from many individuals/timepoints to be pooled, and deconvoluted downstream. This together with the depth of unbiased information generated makes this technology a potential avenue to pursue when investigating mechanism of action in immunotherapy trials.

Up until now, no studies using this technology in the clinical trial setting have yet been published, however, currently a trial is underway. The IMCY-0098 Proof of ACtion in Type 1 Diabetes (IMPACT) trial (clinicaltrials.gov/NCT04524949) will test a proinsulin-derived mimotope in patients with diabetes and use AIM and RNA-Seq to characterize the subsequent immune signature to identify treatment-specific biomarkers. Single cell profiling in the absence of AIM has been used in an antigen agnostic setting; it has been used to examine the immune response in patients with type 1 diabetes after a combined low-dose IL-2 and Treg adoptive cell therapy (patients were given escalating doses of T regs from 3-20 x10^6^/kg). Patients undergoing this treatment had high number of T regs and subsequent gene expression data showed upregulation of functional T reg markers such as *FOXP3, GITR, GARP* and *IKAROS* amongst others (101). A second trial used single cell multiomics to profile PBMCs from patients with long standing diabetes treated with low-dose IL-2 at 3-day intervals at doses ranging from 0.2 to 0.47 × 10^6^ IU/m^2^ and reported an expansion of FOXP3^+^ HELIOS^+^ T regs in treated patients (102).

## Conclusion

Whilst detection of islet specific T cells remains a challenge, the technologies discussed here have the potential to provide a unique insight on the effect of therapies on key players in the pathogenesis of T1D that cannot be obtained using antigen agnostic approaches.

Although challenges remain around some key areas such as the number of cells required and the need for harmonising assays, technological advances mean that multiparametric information derived from a single sample can be used in coordinated efforts to harmonize biomarker discovery and validation (82, 103).

## Author contributions

SA, EW, EP, EC and CD-V performed a review of the literature; SA, EW, EC and CD-V wrote the manuscript. SA conceptualized and supervised the writing of the manuscript. TT provided expertise on aspects of the manuscript and edited. All authors contributed to the article and approved the submitted version.
